# Modulation of torque evoked by wide-pulse, high-frequency neuromuscular electrical stimulation and the potential implications for rehabilitation and training

**DOI:** 10.1038/s41598-021-85645-0

**Published:** 2021-03-18

**Authors:** Chris Donnelly, Jonathan Stegmüller, Anthony J. Blazevich, Fabienne Crettaz von Roten, Bengt Kayser, Daria Neyroud, Nicolas Place

**Affiliations:** 1 Institute of Sport Sciences, Faculty of Biology and Medicine, University of Lausanne, Quartier UNIL-Centre, Building Synathlon, 1015 Lausanne, VD Switzerland; 2 Department of Physical Therapy, University of Florida Health Science Centre, Gainesville, FL USA; 3 Centre for Exercise and Sports Science Research (CESSR), School of Medical and Health Sciences, Edith Cowan University, Joondalup, WA Australia

**Keywords:** Neural circuits, Physiology, Rehabilitation

## Abstract

The effectiveness of neuromuscular electrical stimulation (NMES) for rehabilitation is proportional to the evoked torque. The progressive increase in torque (extra torque) that may develop in response to low intensity wide-pulse high-frequency (WPHF) NMES holds great promise for rehabilitation as it overcomes the main limitation of NMES, namely discomfort. WPHF NMES extra torque is thought to result from reflexively recruited motor units at the spinal level. However, whether WPHF NMES evoked force can be modulated is unknown. Therefore, we examined the effect of two interventions known to change the state of spinal circuitry in opposite ways on evoked torque and motor unit recruitment by WPHF NMES. The interventions were high-frequency transcutaneous electrical nerve stimulation (TENS) and anodal transcutaneous spinal direct current stimulation (tsDCS). We show that TENS performed before a bout of WPHF NMES results in lower evoked torque (median change in torque time-integral: − 56%) indicating that WPHF NMES-evoked torque might be modulated. In contrast, the anodal tsDCS protocol used had no effect on any measured parameter. Our results demonstrate that WPHF NMES extra torque can be modulated and although the TENS intervention blunted extra torque production, the finding that central contribution to WPHF NMES-evoked torques can be modulated opens new avenues for designing interventions to enhance WPHF NMES.

## Introduction

Neuromuscular electrical stimulation (NMES) is an effective tool for neuromuscular rehabilitation and training, with its’ effectiveness being proportional to the evoked torque^1,2^. NMES activates both motor and sensory axons located under the stimulating electrodes, generating contractions through peripheral and/or central (reflexive) pathways^1^. The conventional form of NMES used in clinical settings involves the utilization of short stimulus pulses at low frequencies (pulse duration: < 400 µs, stimulation frequency: 15 to 40 Hz) generating contractions mainly via peripheral pathways^1,3^. A major limitation of these conventional NMES protocols is the strong discomfort associated with the high stimulation intensities necessary to evoke torques that are effective for rehabilitation and training^1^.

Reflexive recruitment of motor units by NMES can be enhanced through the use of wide-pulse, high-frequency (WPHF) stimulation (pulse duration: 1 ms, stimulation frequency: > 80 Hz). The use of wide pulses favors activation of sensory axons as they have a longer strength-duration time constant and a lower rheobase compared with the terminal axonal branches of motoneurons^4^. The use of high stimulation frequencies facilitates the temporal summation of excitatory post-synaptic potentials in spinal motor neurons^5^. Consequently, reflexive activation of motor units via predominantly Ia afferents in the spinal cord^3,6^ is greater with WPHF NMES compared with conventional NMES^7,8^. Using a low constant stimulation intensity to evoke an initial torque of 5–10% maximal voluntary contraction (MVC) torque, WPHF NMES-evoked torques have been reported to reach up to 80% MVC^9^. This progressive increase in WPHF NMES-evoked torque in addition to what would be expected from activation of motor axons through peripheral pathways alone has been termed “extra torque”^7,8^ and occurs in ~ 50% of individuals^10^. This highlights the potential for WPHF NMES to evoke torque levels effective for rehabilitation and training, while overcoming the main limitation of conventional NMES, namely discomfort^11^. Since the effectiveness of NMES protocols is proportional to the evoked torque^12^, there is considerable interest in finding interventions that increase extra torque production to pave the way for WPHF NMES use in clinics. Understanding how modulating the excitability of spinal reflex circuits affects WPHF NMES-evoked torque is needed to optimize these NMES protocols.

The excitability of spinal reflex pathways can be increased and decreased through changes in synaptic input (e.g., presynaptic inhibition of Ia afferents) and the excitability of the motoneuron (e.g. neuromodulation^13^). Two electrical stimulation methods, transcutaneous electrical nerve stimulation (TENS) and direct current stimulation (DCS), can modulate spinal reflex pathways in opposite ways. TENS is a non-pharmacological treatment commonly used for pain management and involves the transcutaneous application of a continuous electrical current (using various intensities and patterns, pulse duration: 50—250 µs, stimulation frequency: 1—200 Hz^14–17^). The sensory volley elicited by high-frequency TENS activates endogenous inhibitory systems in the central nervous system (e.g. inhibitory interneurons and opioid receptors) altering the neural processing of sensory information and providing analgesia during and after stimulation^14–17^. Transcutaneous delivery of DCS to the spinal cord (transcutaneous spinal DCS, tsDCS) can increase activity in reflex pathways during^18,19^ and after stimulation^20–24^ using an anodal but not cathodal electrode configuration. Since WPHF NMES-evoked torque involves, at least partially, motor units recruited reflexively in the spinal cord, the use of TENS or anodal tsDCS during, or before, WPHF NMES may alter WPHF NMES-evoked torque.

The main aim of the present study was to modulate WPHF NMES evoked torque using TENS and tsDCS, two strategies previously shown to affect the state of spinal circuitry *after their application* in opposite ways. Therefore, we applied both interventions before WPHF NMES hypothesizing that high-frequency TENS, through its spinal inhibitory effects, would reduce WPHF NMES-evoked torque by reducing reflexive recruitment of motor units, whilst anodal tsDCS, through its spinal excitatory effects, would increase WPHF NMES-evoked torque.

## Materials

### Participants

Healthy recreationally active participants were recruited to take part to this study. Participants were fully informed of the experimental procedures and risks, before giving written informed consent to participate. The study was performed in accordance with the Declaration of Helsinki and approved by the Research Ethics Committee of the Canton Vaud (2016-00563).

### Experimental design

**Figure 1:**
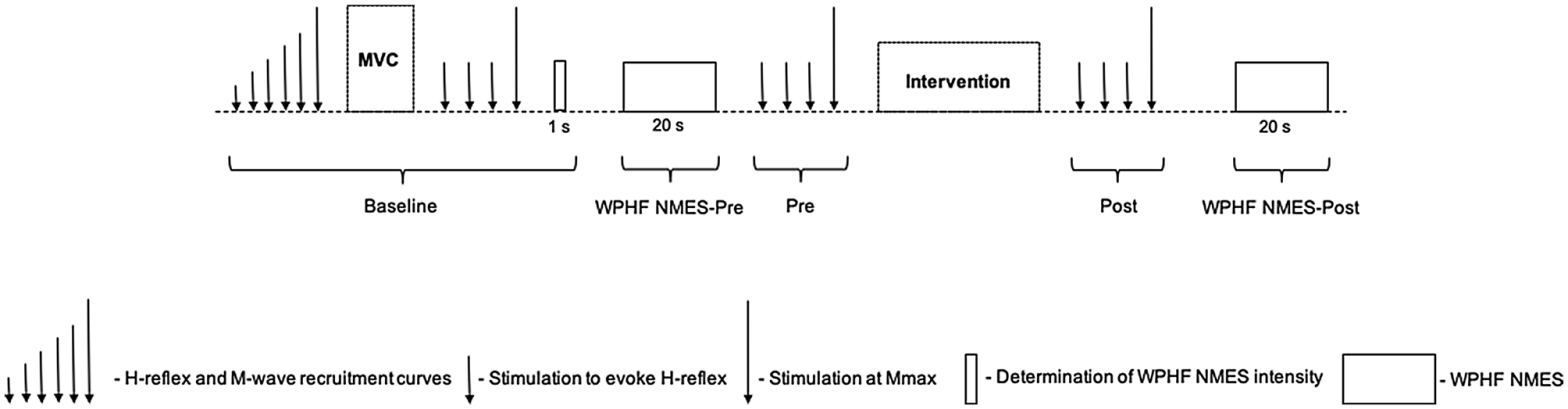
Schematic diagram of experimental design. I_H-reflex_ = stimulation intensity used to elicit a maximal H-reflex. I_Mmax_ = 120% of stimulation intensity used to elicit a maximal M-wave. Stimulation intensity of wide-pulse, high-frequency neuromuscular electrical stimulation (WPHF NMES, stimulation frequency: 100 Hz, pulse duration: 1 ms) was set to produce an initial torque level of 5% maximal voluntary contraction torque. In the transcutaneous electrical nerve stimulation (TENS) experiment, the intervention was high-frequency TENS (TENS trial) or no stimulation (Control trial) delivered over the plantar flexors for 15 min and the bout of WPHF NMES consisted of 3 x 20-s contractions separated by 40-s recovery. In the transcutaneous spinal direct current stimulation (tsDCS) experiment, the intervention was anodal tsDCS (tsDCS trial) or a sham stimulation (Sham trial) delivered over the 11th thoracic vertebrae for 20 min. The bout of WPHF NMES consisted of one contraction.

Two subsets of experiments were performed. Each utilized a randomized crossover study design with its participants taking part in two trials separated by two to seven days. The first set of experiments (TENS experiment) involved ten participants (2 women: mean ± SD, 29 ± 1 years, 167 ± 9 cm, 77 ± 11 kg and 8 men: 27 ± 4 years, 178 ± 5 cm, 71 ± 7 kg) and the second (tsDCS experiment) involved 13 participants (1 woman: 25 years, 168 cm, 56 kg and 12 men: 27 ± 4 years, 178 ± 7 cm, 76 ± 9 kg). The plantar flexor mechanical and electromyographic (EMG) responses to WPHF NMES were assessed before and after each intervention (see Experimental Protocol and Fig. 1). All tests were conducted on the right leg. Participants were requested to refrain from strenuous exercise and caffeine for 24 h before each trial. Participants were first familiarized with all the stimulation modalities used in the experimental trials and were not informed about the potential effects of the two stimulation protocols on WPHF NMES before completion of the study.

### Experimental protocol

A schematic overview of the experimental protocols is shown in Fig. 1. First, *soleus* H-reflex and M-wave recruitment curves were constructed. Single electrical stimulations were delivered to the tibial nerve (see “Nerve stimulation” for details), every 8 s, in 5-mA increments until a visible decrease in the H-reflex amplitude was observed. Subsequently, three stimulations were delivered at each intensity with 2-mA increments over a 20-mA range centered on the intensity eliciting the greatest *soleus* H-reflex amplitude^25^. The current intensity (I) at which the mean *soleus* H-reflex peak-to-peak amplitude was largest (I_H-reflex_) was used for all subsequent stimulations. Single stimulations were then delivered in increments of 10 mA starting at I_H-reflex_ to determine the intensity of maximal stimulation, i.e. until no further increase in peak twitch torque or *soleus* M-wave peak-to-peak amplitude was observed despite a current increase of 20 mA. This intensity was then further increased by 20% (I_Mmax_) to ensure supramaximal stimulation^25^. Participants were then instructed to warm up by performing eight to ten submaximal isometric plantar flexor contractions at 20 – 80% of their estimated MVC torque. They were asked to perform two to three MVCs with the plantar flexors for ∼4 s (no more than 5% variation in torque was tolerated between the two highest MVCs). Participants folded their arms over their chest and were asked to concentrate on exclusively contracting the plantar flexor muscles. Contractions were separated by 1 min of rest. MVC assessment was followed by three stimuli at I_H-reflex_ and one stimulus at I_Mmax_ (Pre-intervention). All electrical stimulations were separated by 8 s. The stimulation intensity required to evoke a torque of 5% MVC (I_WPHF NMES_) was then determined by delivering 1-s WPHF NMES trains (1-ms pulses delivered at 100 Hz) over the *triceps surae* muscles. After 1 min of rest, the first bout of WPHF NMES (WPHF NMES-Pre) was delivered. In the TENS experiment, this consisted of three WPHF NMES contractions with on and off periods of 20 and 40 s, respectively^25^. As the responses to these trains were highly reproducible and analysis of the mean of the three contractions *vs.* only the first contraction gave the same results only one WPHF NMES contraction was evoked in the tsDCS experiment (see “Statistical analysis” section for full details). The stimulation intensity was kept constant during the contraction. Participants were asked to relax and not to voluntarily contract the plantar flexor muscles during the stimulation protocol. Ten seconds after the induced contraction, three stimuli at I_H-reflex_ and one stimulus at I_Mmax_ (Pre) were delivered. Participants remained seated and then received the intervention. In the TENS experiments, TENS (TENS trial) or no stimulation (Control trial) was delivered over the *triceps surae* for 15 min (see “TENS” section for details). In the tsDCS experiments, anodal tsDCS (tsDCS trial) or a sham stimulation (Sham trial) was delivered over the lower thoracic spine for 20 min (see “tsDCS” section for details). The order of intervention and control trials was randomized for each experiment. After 30 s of rest, three stimuli at I_H-reflex_ and one stimulus at I_Mmax_ were delivered (Post) before another 20-s WPHF NMES (WPHF NMES-Post).

### Torque recordings

The torque produced by the plantar flexors was measured with an instrumented pedal equipped with a strain gauge sensor (capacity: 110 N m, Vishay Micro Measure, Raleigh, NC). Participants sat on a stool with knee, ankle, and hip angles set at 90° and their foot securely strapped to the ergometer at the level of the ankle and metatarsus. To limit the contribution of muscle groups other than the plantar flexors, the thigh was clamped down to the stool proximal to the knee. The torque signal was recorded at 1250 Hz using an analog–digital conversion system (MP150, BIOPAC, Goleta, USA).

### Electrical stimulation

#### Nerve stimulation

Electrical nerve stimulation was delivered to the tibial nerve transcutaneously by a cathode self-adhesive electrode (1-cm diameter, Meditrace 100, Tyco, Markham, Canada) placed in the popliteal fossa over the tibial nerve and an anode (10 × 5 cm, Compex, Ecublens, Switzerland) located over the anterior surface of the knee, 2 to 3 cm proximal to the patella. The optimal position of the cathode was determined using a hand-held cathode ball electrode (0.5-cm diameter, Compex, Ecublens, Switzerland). Once determined, the cathode was firmly fixed to this site using a Velcro strap. Rectangular-wave pulses (1-ms duration) were generated by a high-voltage (max 400 V) constant-current stimulator (Digitimer DS7AH, Hertfordshire, UK) and delivered at 400 V as single electrical stimuli.

#### WPHF NMES

WPHF NMES was delivered transcutaneously via two 10 × 5-cm rectangular self-adhesive surface electrodes (Uni-Patch Minnesota, USA). The anode was placed over the *gastrocnemii* (~ 4 cm distal to the popliteal fossa) and the cathode over the *soleus* (~ 10 cm proximal to the calcaneus). Pulses were delivered by a second constant-current stimulator (Digitimer DS7AH, Hertfordshire, UK), with a 1-ms pulse duration delivered at 400 V, with WPHF NMES delivered at 100 Hz. Muscle stimulation was chosen since the intra-individual test-retest reliability of WPHF NMES-evoked forces delivered over the *triceps surae* muscle belly is very good (intraclass correlation coefficient >0.95^9^). In addition, extra force generation has been shown to be similar between nerve and muscle stimulation using an initial evoked torque of 5% MVC torque, while the discomfort associated with muscle stimulation is approximately half of that associated with nerve stimulation^9^.

#### TENS

A TENS unit (Compex Wireless, Ecublens, Switzerland) was used to deliver TENS via two 10 × 5-cm rectangular self-adhesive surface electrodes (Compex, Ecublens, Switzerland). The location on the *triceps surae* was identical to that of WPHF NMES. High-frequency TENS^26^ was delivered continuously for a period of 15 min, with a constant pulse duration of 50 µs and a frequency varying from 150 to 50 Hz over 2 s followed by variation from 50 to 150 Hz over 2 s. Once the TENS unit was switched on, participants were told to report the onset of any ‘tingling’ or ‘buzzing’ sensations beneath the electrodes. The TENS current intensity was then increased until subjects reported a ‘strong but comfortable'’ sensation without any visible muscle contraction (i.e. below motor threshold;^26–28^). After 7.5 min, participants were asked whether the reported sensation had decreased. If so, the intensity was increased but still kept below motor threshold.

#### tsDCS

A battery-powered stimulator (Starstim, Neuroelectrics, Barcelona, Spain) was used to deliver anodal tsDCS via two 5 x 7 cm saline (0.9%) soaked sponges. The anode (stimulation electrode) was placed on the spine at the level of the 11th thoracic vertebra^18–20^ and the cathode (return electrode) under the right shoulder blade at the level of the posterior deltoid^21,29^. The tsDCS stimulation protocol lasted 20 min and was delivered with a current of 2 mA. There was a progressive increase and decrease in current intensity over the first and last 30 s, respectively. In the sham protocol, tsDCS was active only during the first and last 30 s to simulate the stimulation since the initial tingling sensation felt by the participants disappeared regardless of whether the stimulation is maintained or deactivated^18^. The level of impedance was monitored regularly during stimulation^18,29^.

#### EMG recordings

Resistance between pairs of electrodes was minimized by shaving and cleaning the skin with alcohol. EMG activity was recorded from the *soleus* with pairs of circular silver chloride (Ag/AgCl) (1-cm recording diameter) self-adhesive electrodes (Meditrace 100, Tyco, Markham, Canada) with an inter-electrode (center-to-center) distance of ~2 cm, according to SENIAM recommendations^30^. The electrodes were placed lengthwise over the *soleus* muscle belly at a distance corresponding to two thirds of the distance between the medial condyle of the femur and the medial malleolus. The reference electrode was placed over the ipsilateral patella. The positions of all EMG and stimulation electrodes were marked with indelible ink to allow identical repositioning. EMG signals were amplified with a gain of 1000, filtered with a bandwidth frequency between 10 and 500 Hz, digitized at a sampling frequency of 5 kHz, and recorded with the analog–digital conversion system. EMG and torque data were stored and analyzed offline with commercially available software (Acqknowledge, BIOPAC Systems, Goleta, USA).

### Data analysis and presentation

#### Torque data

Isometric MVC torque was considered as the highest torque attained during the MVC trials. The WPHF NMES-evoked torque was quantified in the 2nd (initial torque) and 20th (final torque) seconds of stimulation. As in previous studies^7,8,10,31,32^, “extra torque” was calculated as the difference between the final and initial torques (extra torque = final torque – initial torque). Extra torque was taken as an indication of a “warm-up” effect caused by the stimulation^33^ and indirect evidence of motor units being recruited through the central pathway. The torque-time integral (TTI, the area under the torque-time trace) was also quantified for each WPHF NMES contraction^10,25^ from the onset of evoked torque until the torque returned to resting values.

#### EMG data

Peak-to-peak amplitude was used to quantify H-reflex and M-wave responses to single electrical stimuli. For H reflex, the mean of the three responses at each time point was used for analysis. The H-reflex was normalized by M_max_ and the H-reflex-to-M_max_ ratio (H-reflex/M_max_) was used to evaluate the balance between facilitation and inhibition from pre-synaptic inhibitory input at the spinal level^34^ as per previous WPHF NMES studies e.g.^10^. We chose this approach over assessing the H-reflex recruitment curve as we wanted to limit the time from the end of the intervention to the start of WPHF NMES.

The EMG signals recorded during MVCs were quantified as root mean square (RMS) amplitudes (symmetric 50-ms moving average) over a 500-ms interval around MVC torque (250 ms before and after MVC torque) for the *soleus*. *Soleus* RMS EMG amplitude was also quantified over a 500-ms interval from 210 ms after the final stimulation artefact (to avoid the step response of the EMG filter) for each WPHF NMES contraction (i.e. after cessation of electrical activation of the muscle, and without voluntary drive to the muscle) and normalized to the RMS amplitude of the maximal EMG activity during the highest MVC to assess sustained EMG activity^25^. The presence of sustained EMG activity was used to indirectly highlight the presence of PICs^25,33^ and also to represent indirect evidence of motor units being recruited through the central pathway.

### Statistical analysis

Data were plotted in graphical format to assess sampling distributions using GraphPad Prism for Mac (GraphPad Software 7, Inc., USA). Data were not normally distributed and therefore non-parametric statistical tests were used. Statistical significance was set at an alpha level of *P* < 0.05.

In the TENS experiment, Friedman ANOVAs were performed to test for differences in TTI, extra torque and sustained EMG activity between the three WPHF NMES trains within a bout of WPHF stimulation (WPHF NMES-Pre). The TTI (Control, *P* = 0.23; TENS, *P* = 0.19), extra torque (Control, *P* = 0.44; TENS, *P* = 0.97) and sustained EMG amplitude (Control, *P* = 0.19; TENS, *P* = 0.44) were similar for the three WPHF NMES trains in each bout of WPHF NMES. In addition, the intra-individual reliability of these trains was evaluated by means of intraclass correlation coefficient (ICC; two-way mixed effects, absolute agreement^35^). In agreement with^9^, the ICC was very high for all parameters: TTI (Control, ICC = 0.93; TENS, ICC = 0.96), extra torque (Control, ICC = 0.97; TENS, ICC = 0.99) and sustained EMG amplitude (Control, ICC = 0.96; TENS, ICC = 0.88). Therefore, the mean TTI, extra torque and sustained EMG amplitude data from the three WPHF NMES trains in each bout of WPHF NMES were used for subsequent analyses in the TENS experiment and only one WPHF NMES contraction was subsequently evoked in the tsDCS experiment.

For each experiment, Wilcoxon signed rank tests were performed to test for baseline differences between trials in MVC torque, stimulation intensities (I_H-reflex,_ I_Mmax_, I_WPHF NMES_), H-reflex/M_max_ ratio, TTI, initial torque, extra torque and sustained EMG amplitude. To assess whether extra torque production occurred in each experiment, the initial and final torques at WPHF NMES-Pre were compared using Wilcoxon signed rank tests. For these analyses, the mean value from the WPHF NMES-Pre contractions (6 contractions for the TENS experiment and 2 contractions for the tsDCS experiment) for each participant were used. Effect sizes (ES) were calculated for the differences in initial and final torque in each experiment by dividing the Z-score by the $\surd$ N. ES of 0.1, 0.3 and > 0.5 were interpreted as small, medium and large respectively^36^.

Wilcoxon signed rank tests were also performed to test for changes in TTI, extra torque and sustained EMG amplitude under control conditions (i.e. control trial in the TENS experiment and the sham trial in the tsDCS experiment). In both experiments there were no changes in TTI (Control, *P* = 0.19; Sham, *P* = 0.68), extra torque (Control, *P* = 0.92; Sham, *P* = 0.21) or sustained EMG amplitude (Control, *P* = 0.62; Sham, *P* = 0.60) from WPHF NMES-Pre to WPHF NMES-Post under control conditions.

Therefore, for each experiment, differences between trials in the changes in TTI, extra torque, sustained EMG amplitude (change = WPHF NMES-Post – WPHF NMES-Pre) and H-reflex/M_max_ ratio (change = Post-Pre) were also tested using Wilcoxon signed rank tests (see Fig. 1 for time-points). ES were calculated for the changes in TTI, extra torque, sustained EMG activity and H-reflex/M_max_ ratio in each experiment as described above. Spearman’s correlation coefficients (r_s_) were computed between changes in TTI and the changes in extra torque and sustained EMG amplitude for each trial. Data were analyzed using GraphPad Prism for Mac (version 7, GraphPad Software, Inc., USA) and effect sizes were calculated using R. Finally, “responders” were identified as a participant with a positive ET in both trials (intervention and control) within an experiment at WPHF NMES-Pre. Statistical comparisons in the TENS experiment were performed on N = 10 and in the tsDCS experiment N = 13. Data in text are presented as median (25th percentile to 75th percentile).

## Results

There were no differences between trials at baseline for any variable tested (Table 1). In both experiments, there were no differences between trials for the initial WPHF NMES-Pre torque. In the TENS experiment, initial torque was 4 (3 to 5)% MVC torque in the control trial and 4 (3 to 7)% MVC torque in the TENS trial (*P* = 0.32). In the tsDCS experiment, initial torque was 5 (4 to 6)% MVC torque in the control trial and 5 (3 to 7)% MVC torque in the tsDCS trial (*P* = 0.42). Comparison of the initial and final torques evoked at WPHF NMES-Pre showed a significantly higher final torque in the TENS experiment (*P* = 0.037, ES = 0.66, Fig. 4A). There was no difference between initial and final torques in the tsDCS experiment (*P* = 0.99, ES = 0.01, Fig. 4D).

**Table 1:** Baseline variables in the TENS and tsDCS experiments.

	TENS experiment			tsDCS experiment		
	TENS trial	Control trial	*P*	tsDCS trial	Sham trial	*P*
MVC torque (N m)	158 (146 to 172)	172 (157 to 173)	0.32	165 (155 to 183)	182 (156 to 187)	0.41
H-reflex/M_max_ (%)	50 (37 to 54)	48 (28 to 52)	0.09	35 (25 to 42)	28 (12 to 39)	0.08
I_H-reflex_ (mA)	32 (23 to 46)	35 (25 to 46)	0.63	33 (14 to 44)	23 (20 to 31)	0.72
I_Mmax_ (mA)	125 (120 to 159)	132 (111 to 153)	0.71	168 (120 to 174)	138 (120 to 168)	0.35
I_WPHF NMES_ (mA)	12 (8 to 19)	18 (9 to 24)	0.20	13 (10 to 14)	12 (10 to 13)	0.66

Values are median (25th percentile to 75th percentile).

*TENS* transcutaneous electrical stimulation, *tsDCS* transcutaneous spinal direct current stimulation, *MVC* maximal voluntary contraction, *H-reflex* H-reflex peak-to-peak amplitude, *M*_*max*_ maximal M-wave peak-to-peak amplitude, *I*_*H-reflex*_ stimulation intensity used to elicit a maximal H-reflex, *I*_*Mmax*_ 120% of stimulation intensity used to elicit a maximal M-wave, *I*_*WPHF NMES*_ wide-pulse, high-frequency neuromuscular electrical stimulation (WPHF NMES) intensity.

### TENS intervention

**Figure 2:**
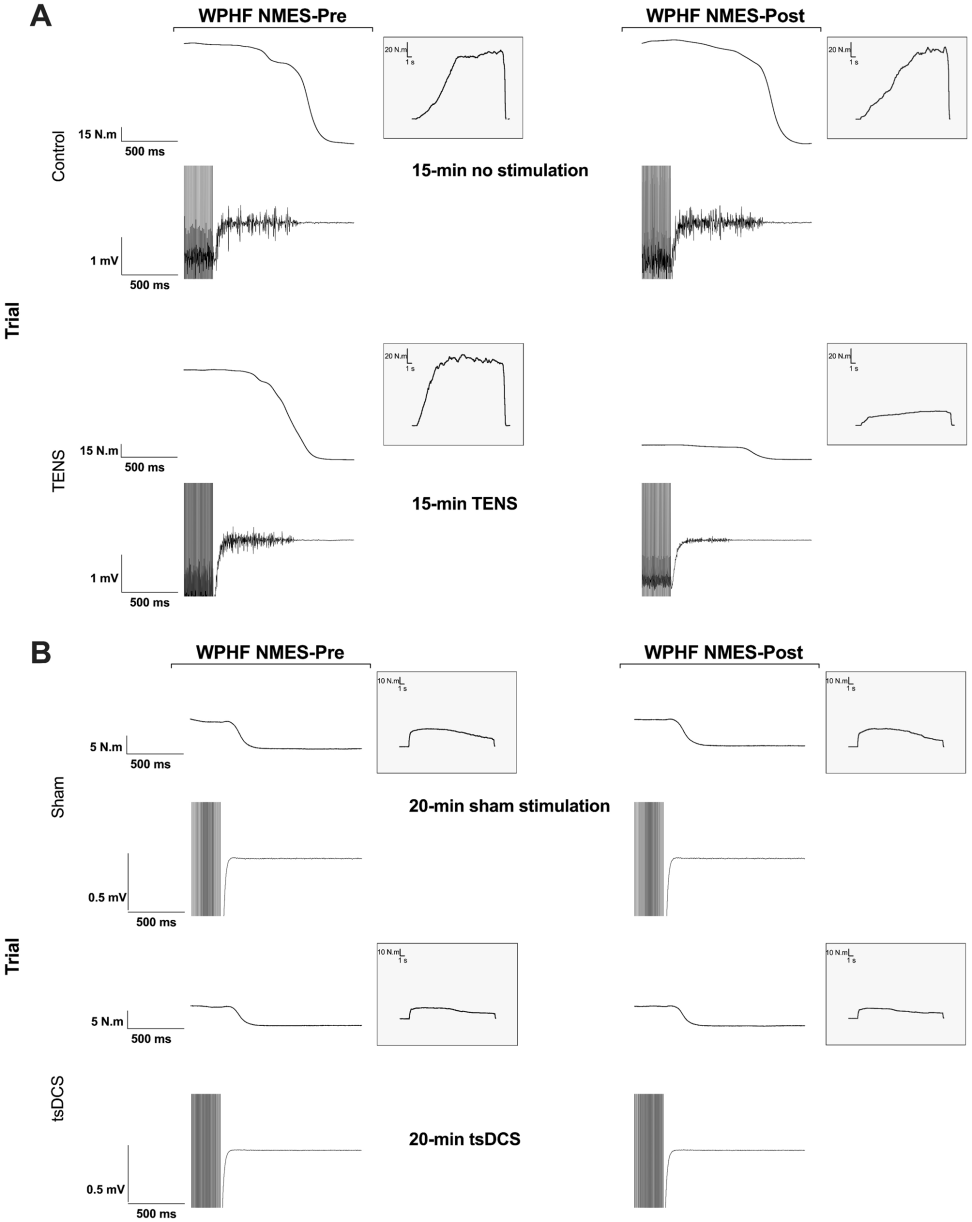
Original WPHF NMES-evoked torque and EMG traces for one participant presented before (WPHF NMES-Pre) and after (WPHF NMES-Post) **(A)** a 15-min period of TENS (TENS trial) or no stimulation (Control trial) and **(B)** a 20-min period of tsDCS (tsDCS trial) or sham stimulation (Sham trial). Each window lasts 1500 ms and includes the final 26 stimulation artefacts of each 20-s WPHF NMES. The insert shows the complete WPHF NMES (20-s stimulation) force trace. In the TENS experiment **(A)** compared to the control condition, WPHF NMES-evoked torque and sustained EMG activity were reduced following a 15-min period of TENS. In the tsDCS experiment **(B)** there was no increase in WPHF NMES-evoked torque or sustained EMG activity following a 20-min period of tsDCS or sham stimulation.

**Figure 3:**
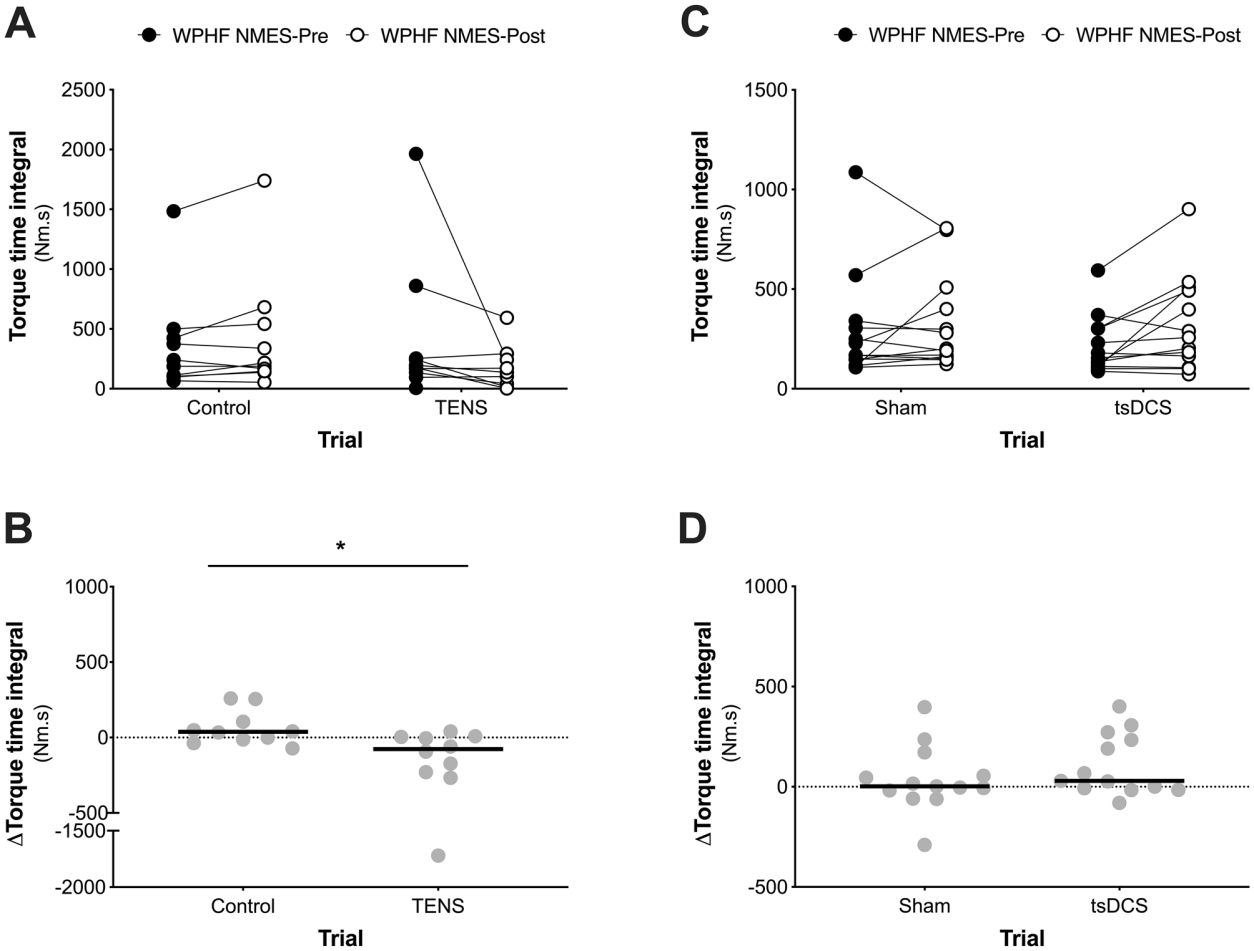
**(A)** Torque-time integral (TTI) presented before (WPHF NMES-Pre) and after (WPHF NMES-Post) a 15-min period of TENS (TENS trial) or no stimulation (Control trial). **(B)** Change in the TTI after a 15-min period of TENS (TENS trial) or no stimulation (Control trial). **(C)** TTI presented before (WPHF NMES-Pre) and after (WPHF NMES-Post) a 20-min period of tsDCS (tsDCS trial) or sham stimulation (Sham trial). **(D)** Change in the TTI after a 20-min period of tsDCS (tsDCS trial) or sham stimulation (Sham trial). Data are presented as individual data points and the line represents the median. *Significant difference between trials (*P* = 0.001). In TENS experiment **(A,B)** a significant reduction in the torque-time integral was observed when wide-pulse, high-frequency NMES stimulations were imposed after a bout of TENS compared to the control condition. In tsDCS **(C,D)** no significant change in the torque-time integral was observed when a wide-pulse, high-frequency NMES stimulation was imposed after a bout of tsDCS compared to the sham condition.

**Figure 4:**
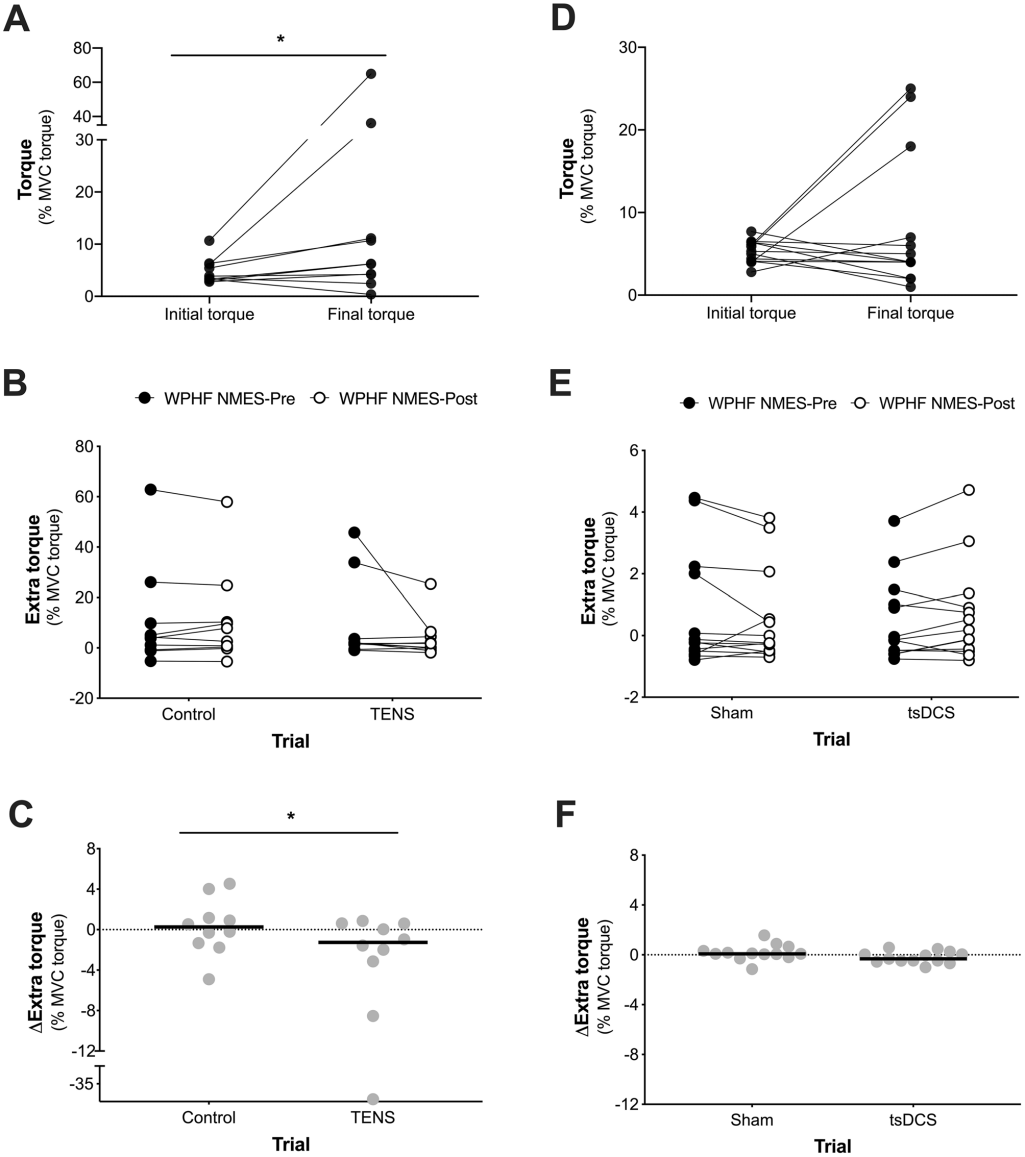
**(A)** Initial and final torque during WPHF NMES presented for the mean of all WPHF NMES-Pre contractions in the TENS experiment. **(B)** Extra torque presented before (WPHF NMES-Pre) and after (WPHF NMES-Post) a 15-min period of TENS (TENS trial) or no stimulation (Control trial). **(C)** Change in extra torque after a 15-min period of TENS (TENS trial) or no stimulation (Control trial). **(D)** Initial and final torque during WPHF NMES presented for the mean of all WPHF NMES-Pre contractions in the tsDCS experiment. **(E)** Extra torque presented before (WPHF NMES-Pre) and after (WPHF NMES-Post) a 20-min period of tsDCS (tsDCS trial) or sham stimulation (Sham trial). **(F)** Change in extra torque after a 20-min period of tsDCS (tsDCS trial) or sham stimulation (Sham trial). Data are presented as individual data points and the line represents the median. *Significant difference between trials (**A**: *P* = 0.037, **C**: *P* = 0.013). In TENS experiment **(A–C)** a significant reduction in extra torque was observed when wide-pulse, high-frequency NMES stimulations were imposed after a bout of TENS compared to the control condition. In the tsDCS experiment **(D–F)** no significant change in extra torque was observed when a wide-pulse, high-frequency NMES stimulation was imposed after a bout of tsDCS compared to the sham condition.

**Figure 5:**
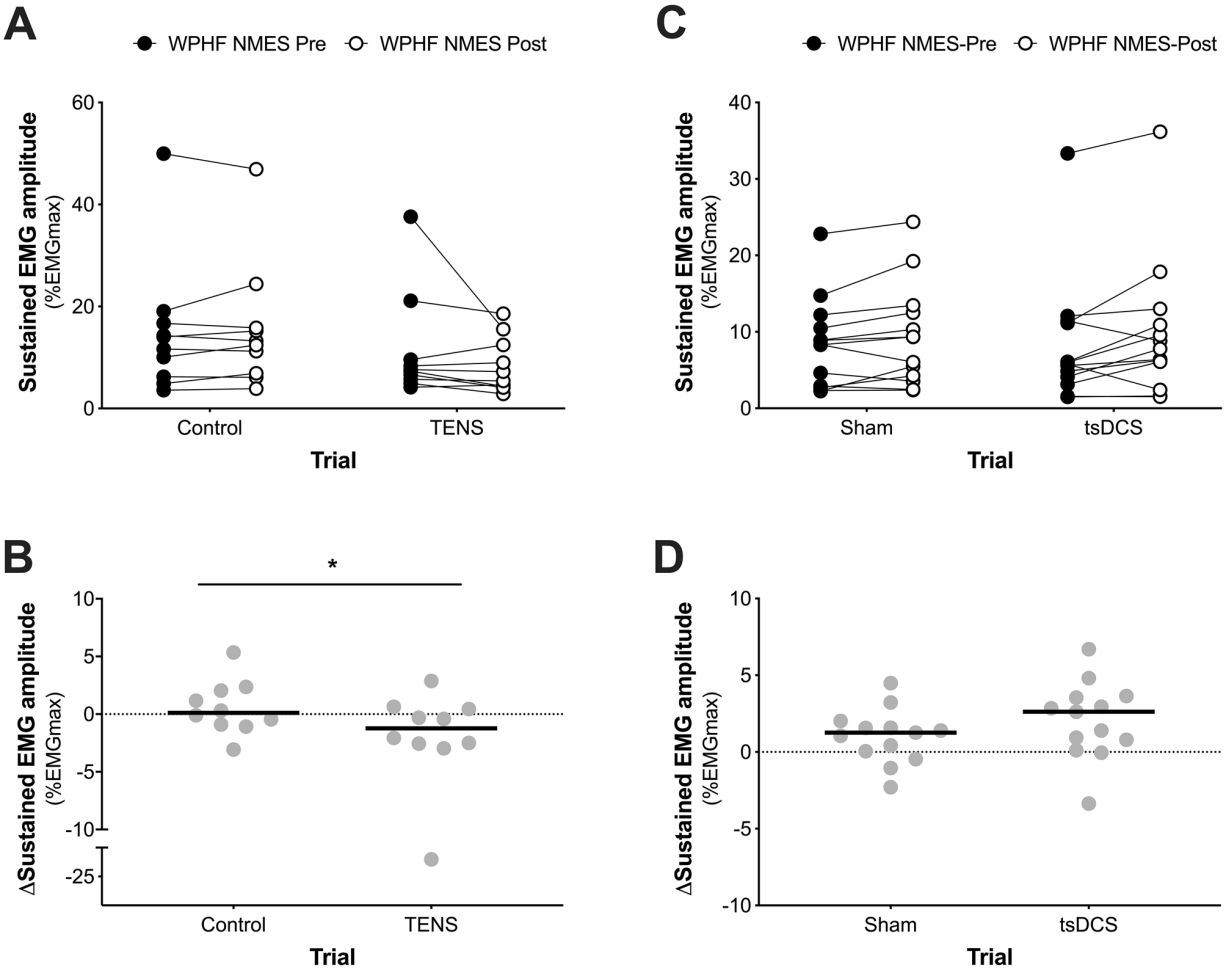
**(A)** Sustained EMG amplitude recorded before (WPHF NMES-Pre) and after (WPHF NMES-Post) a 15-min period of TENS (TENS trial) or no stimulation (Control trial). **(B)** Change in sustained EMG amplitude after a 15-min period of TENS (TENS trial) or no stimulation (Control trial). **(C)** Sustained EMG amplitude recorded before (WPHF NMES-Pre) and after (WPHF NMES-Post) a 20-min period of tsDCS (tsDCS trial) or sham stimulation (Sham trial). **(D)** Change in sustained EMG amplitude recorded after a 20-min period of tsDCS (tsDCS trial) or sham stimulation (Sham trial). Data are presented as individual data points and the line represents the median. *Significant difference between trials (*P* = 0.005). A significant reduction in EMG activity was observed in the period immediately after the cessation of wide-pulse, high-frequency NMES stimulations when imposed after a bout of TENS compared to the control condition **(A,B)**. In the tsDCS experiment **(C,D)** no significant change in EMG activity was observed in the period immediately after the cessation a wide-pulse, high-frequency NMES stimulation was imposed after a bout of tsDCS compared to the sham condition.

**Figure 6:**
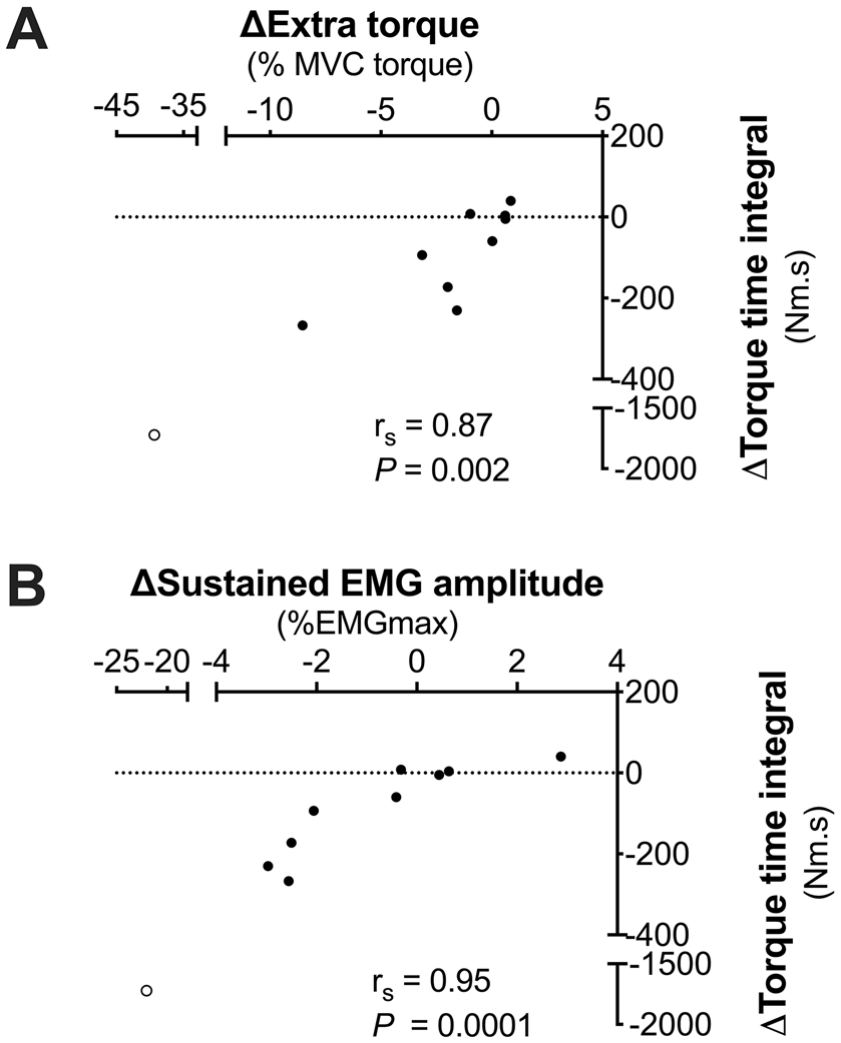
Relationship between changes in the torque-time integral (TTI) and **(A)** extra torque and **(B)** sustained EMG amplitude following a 15-min period of TENS. Extreme value (included in analysis) is represented by an unfilled circle. Removing this participant from the analysis did not affect the conclusion. Significant, negative (Spearman’s) correlations were observed; no correlation was detected in the control condition.

In the TENS experiment, no differences were observed between trials for TTI (*P* = 0.92, Fig. 3A), extra torque (*P* = 0.92, Fig. 4B) or sustained EMG amplitude (*P* = 0.12, Fig. 5A) at WPHF NMES-Pre. Five of the nine participants increased the TENS current intensity after 7.5 min [0–7.5 min = 56 (51 to 65) mA; 7.5–15 min = 58 (56 to 70) mA]. Raw torque traces and EMG recordings from a single participant before and after a 15-min period of TENS or no-stimulation Control are shown in Fig. 2A. Compared to Control, WPHF NMES-evoked torque and sustained EMG amplitude were reduced following the 15-min period of TENS, with significant between-trial differences for changes in TTI (*P* = 0.001, ES = 0.79, Fig. 3B), extra torque (*P* = 0.013, ES = 0.76, Fig. 4C) and sustained EMG amplitude (P = 0.005, ES = 0.82, Fig. 5B). In the TENS trial, there was a significant negative relationship between the change in TTI and changes in both extra torque (rs = 0.87, P = 0.002, Fig. 6A) and sustained EMG amplitude (rs = 0.95, P = 0.0001, Fig. 6B). There were no significant relationships between the change in TTI and the change in extra torque (r_s_ = 0.27, *P* = 0.45) or change in sustained EMG amplitude (r_s_ = 0.28, *P* = 0.43) in Control. There was no significant difference in the change in H-reflex/M_max_ ratio between trials [*P* = 0.142, ES = 0.54; Control trial: Pre = 48 (35 to 58)% vs. Post = 49 (25 to 61)%, TENS trial: Pre = 51 (34 to 62)%, Post = 45 (25 to 59)%].

### tsDCS intervention

In the tsDCS experiment, there were also no differences between trials for WPHF NMES-Pre TTI (*P* = 0.38, Fig. 3C), extra torque (P = 0.41, Fig. 4E) or sustained EMG amplitude (P = 0.54, Fig. 5C). Raw torque traces and EMG recordings from a single participant before and after a 20-min period of tsDCS or Sham are shown in Fig. 2B. Compared to Sham, WPHF NMES-evoked torque and sustained EMG amplitude were not different following a 20-min period of tsDCS, with no between-trial differences in the changes in TTI (P = 0.19, ES = 0.38, Fig. 3D), extra torque (P = 0.08, ES = 0.49, Fig. 4F) or sustained EMG amplitude (P = 0.06, ES = 0.53, Fig. 5D). In the tsDCS and Sham trials, no significant relationships were observed between the change in TTI and changes in extra torque (tsDCS trial: r_s_ = 0.38, *P* = 0.20, Sham Trial: r_s_ = 0.16, *P* = 0.60) or sustained EMG amplitude (tsDCS trial: r_s_ = − 0.23, *P* = 0.45, Sham Trial: r_s_ = − 0.3, *P* = 0.33). There was no significant difference in the change in H-reflex/M_max_ ratio between trials [*P* = 0.56, ES = 0.20; Control trial: Pre = 33 (25 to 44) % vs. Post = 37 (26 to 46) %, TENS trial: Pre = 32 (22 to 49) %, Post = 31 (28 to 47) %].

## Discussion

The aim of the present study was to investigate the influence of high-frequency TENS and transcutaneous spinal DCS (tsDCS) on the mechanical and EMG responses to wide-pulse, high-frequency (WPHF) NMES. Our first hypothesis was that high-frequency TENS would reduce WPHF NMES-evoked torque due to reduced reflexive recruitment of motor units, and our results support this hypothesis (Figs. 3B, 4C, 5B). This reduction in WPHF NMES-evoked torque was associated with a reduction in muscle activity detected after the cessation of NMES (and without voluntary muscle contraction), i.e. less ‘sustained EMG amplitude’, which is consistent with reduced reflexive motor unit recruitment (Fig. 6A). It was also associated with reduced extra torque during the 20-s WPHF NMES period itself, which is consistent with fewer motor units being recruited through central pathways (Fig. 6B). The changes in evoked torque occurred without change in the H-reflex/M_max_ ratio. Our second hypothesis was that tsDCS would increase the torque evoked by WPHF NMES. However, anodal tsDCS had no effect on the evoked torque, sustained EMG activity or the H-reflex/M_max_ ratio. Overall, our findings support the concept that reflexive recruitment of motor units is a key factor underpinning WPHF NMES-evoked torque but does not indicate that the tsDCS protocol used would be effective in a clinical environment.

WPHF NMES recruits motor units through both peripheral and central (reflexive) pathways. Irrespective of the pathways underpinning WPHF NMES-evoked torque, our results show that only TENS (not anodal tsDCS) modulated the torque evoked by WPHF NMES (− 50% compared to control). TENS applied in the periphery activates large diameter afferent fibers and in turn endogenous inhibitory systems in the central nervous system^17^. In support of this contention (i) blocking cutaneous afferents with lidocaine does not affect TENS mediated analgesia^37^, (ii) TENS applied to the contralateral “mirror side” reverses hyperalgesia bilaterally^38^, and (iii) TENS provides analgesia outside of the area of application^39^. As such our results are most likely due to the effect of TENS at the spinal cord level. Electrophysiological studies performed in animal models demonstrate that high-frequency TENS increases gamma-aminobutyric acid (GABA, the major inhibitory neurotransmitter) concentration in the spinal cord through activation of opioid receptors^15^. Furthermore, blockade of GABA_A_ receptors in the spinal cord prevents the analgesic effect of high-frequency TENS^15^. The opioid- and GABA-mediated analgesia provided by TENS has since been confirmed in humans using naloxone, an opioid antagonist^16^. Thus, a possible explanation for the reduced WPHF NMES-evoked torque may be alterations in reflexive recruitment resulting from TENS inhibitory effects on spinal circuits.

Two measures were made to assess reflexive recruitment of motor units by WPHF NMES: extra torque and sustained EMG activity. Extra torques observed during electrical stimulation of afferents and motor nerves in humans (typically observed in 40-60% participants^9,10,32^ and 60% and 38% in the TENS and tsDCS experiments, respectively) as well as the subsequent sustained EMG activity have been attributed to a combination of temporal summation of post-synaptic potentials^5^ and persistent inward current (PIC)-mediated warm-up and self-sustained motoneuron firing^7,8,31,40^. The temporal summation of excitatory post-synaptic potentials (primarily generated by the neurotransmitter glutamate^41^) occurs when multiple stimuli are delivered at high-frequency, i.e. the inter-stimulus interval is shorter than excitatory post-synaptic potential duration^42^. The size and duration of excitatory post-synaptic potentials are influenced by both excitatory and inhibitory inputs^43^. One inhibitory input is conveyed by GABA, which in rats has been demonstrated to reduce the Ia-motoneuron excitatory post-synaptic potential amplitude^44^. PICs are generated by voltage-sensitive Na^+^ and Ca^2+^ currents that amplify and prolong the effects of synaptic (ionotropic) input, and have marked characteristics such as wind-up and self-sustained firing^43^. The control of PICs by neurotransmitters is powerful and the motor output generated by a given pattern of ionotropic input can vary by up to five-fold^43,45^, similar in magnitude to the range of WPHF NMES-evoked torque previously reported^8,32,46^. Specifically, norepinephrine (NE) and serotonin (5-HT) facilitate, whereas GABA suppresses, the generation of PICs^45,47,48^ allowing for modulation of the response of a motoneuron to a given synaptic input. Indeed, *in vitro* pharmacological studies using bicuculline and baclofen, selective agonists of GABA_A_ and GABA_B_ receptors, respectively, have shown that GABA action can inhibit the stable membrane potentials above the resting membrane potential that are generated by PICs, i.e. plateau potentials^45^. Thus, lower WPHF NMES-evoked torque following TENS might be mediated by reduced temporal summation of excitatory post-synaptic potentials and/or PIC activation. Further in support of a spinal contribution to WPHF NMES-evoked torque through modulation of post-synaptic potentials and PICs, reduced WPHF NMES-evoked torque following TENS was independent of changes in the H-reflex/M_max_ ratio. This result was expected as TENS has been reported to not affect the H-reflex amplitude^49^. These findings are consistent with the fact that summation of excitatory post-synaptic potentials is only achieved by consecutive inputs^50^ and PICs take time to “wind up” in response to synaptic inputs^51^. Hence single stimuli, as used in H-reflex measurements, are unlikely to activate PICs.

Overall, several lines of evidence suggest that the reduced WPHF NMES-evoked torque following TENS is likely to be mediated by an increased spinal inhibition, consequently affecting reflexive activation of motor units involving temporal summation of post-synaptic potentials, PIC generation and/or PIC strength. Together these data (Figs. 3B, 4C, 5B, 6A,B) further support the concept of a spinal contribution to WPHF NMES-evoked torque. The notion that temporal summation of excitatory post-synaptic potentials and/or PICs amplify the motor output for a given pattern of synaptic input may explain why we observed a much larger reduction in WPHF NMES-evoked torque (~ 50%) than sustained EMG activity (~ 10%), as a reduction in synaptic input and its amplification (the PIC strength) would result in a striking reduction in evoked torque due to reduced motor unit activation. Furthermore, the changes in extra torque and sustained EMG amplitude (indirect evidence of PICs) and the changes in TTI after TENS were highly correlated (Fig. 6). Despite only being associative evidence, the result suggests that reflexive recruitment is important for WPHF NMES-evoked torque and that the reduction in WPHF NMES-evoked torque following TENS might be mediated by the inhibitory actions of GABA in the spinal cord, leading to a reduction in excitatory post-synaptic potentials and/or inhibition of PIC generation. Thus, we suggest that TENS for pain relief should not be applied before WPHF NMES for rehabilitation.

In the tsDCS trial there were also no changes in the H-reflex/M_max_ ratio, consistent with previous studies in which anodal tsDCS applied for 15 min at the 11th thoracic vertebrae did not alter the *soleus* maximal H-reflex amplitude/M_max_ ratio^20,52^. In contrast, Winkler, et al.^20^ reported that anodal tsDCS induced a lasting decrease in H-reflex post-activation depression, which may alter excitatory post-synaptic potentials. Despite this, there was no effect of tsDCS on WPHF NMES-evoked torque, extra torque or sustained EMG activity. In light of the evidence suggesting that TENS alters WPHF NMES-mediated PIC activation/strength and in turn evoked torque, one tentative explanation for no effect of tsDCS on these parameters could be that it did not affect PICs and the gain of synaptic input^53^, as evidenced in the present study by the lack of change in extra torque and sustained EMG activity. The effect of transcranial DCS has been shown to be highly variable between individuals^54^ (e.g. in the present study, TTI was greater after tsDCS than Sham in 7/13 participants) and our results cannot provide final explanations for the differing effects of TENS and tsDCS on WPHF NMES-evoked torque. We acknowledge that in our study the number of “responders” to WPHF NMES was lower in the tsDCS experiment compared with the TENS experiment, and extra torque at baseline at the group level was only observed in the later condition. It remains to be investigated whether the effect of tsDCS on WPHF NMES-evoked torque would be beneficial in a group of “responder” participants to improve extra torque production. Overall, studies with larger sample sizes and performing separate analyses of “responders” and “non-responders” to WPHF NMES may bring clarification.

Although it appears likely that changes in spinal processing of sensory information provides the most plausible explanation for the reduction in WPHF NMES-evoked torque following TENS, our data do not allow for a comprehensive explanation to be provided. The notion that WPHF NMES-evoked torque is influenced by peripheral mechanisms is consistent with our finding of reduced, but not abolished, sustained EMG activity and evoked-torque following TENS. Indeed, previous studies^55,56^ show that the recruitment of motor units via peripheral pathways and intramuscular mechanisms also contribute to WPHF NMES-evoked torque. Furthermore, the torque evoked by 10 to 20 s of WPHF NMES with a constant stimulation intensity varies considerably between individuals^10,25,32^. In accordance with previous studies using a similar stimulation pattern which reported inter-individual variation of ~150% in WPHF NMES-evoked torque^10,25^, we observed a large inter-individual variability in both trials with a CV of ~130%. In contrast intra-individual variation has been reported to be much lower (~ 20%;^9^). Indeed, the median percentage change in WPHF NMES-evoked torque from Pre to Post in the TENS trial was greater (~ − 50%) than this intra-individual variation. In the tsDCS trial, the non-significant median change was ~ − 14%. Given that the initial torque evoked by WPHF NMES was similar between trials and experiments, we are confident that our conclusion that TENS reduces, whereas tsDCS has no effect on, WPHF NMES-evoked torque is correct. However, whether the effects of each intervention are different in responders compared with non-responders to WPHF NMES remains to be investigated.

In summary, we show, for the first time, that WPHF NMES-evoked torque can be modulated by a preceding intervention. This conclusion is based on the observed reductions in extra torque and sustained EMG activity in response to WPHF NMES when preceded by TENS, while the anodal tsDCS protocol we used had no effect. Whereas Ia afferent input, summation of post-synaptic potentials and PICs seem to be the most likely physiological phenomenon underpinning WPHF NMES-evoked torque, modulation of WPHF NMES-evoked torque by TENS was independent of changes in the H-reflex/M_max_ ratio, i.e. an index of the balance between inhibitory and excitatory input at the spinal cord. This result thus suggests the possibility that the central contribution to evoked torques might be *increased* by an intervention that enhances sensory input or motoneuron excitability. Given the potent effects of 5-HT and NE on PICs, it is interesting to consider the possibility of altering their concentrations in the spinal cord using drugs such as reuptake inhibitors^57^. Alternatively, other electrical stimulation protocols such as low-frequency TENS (known to increase serotonin levels in the spinal cord^58^), sensory stimulation e.g.^59^ or optimizing other DCS protocols e.g.^54^ could be considered. Finally, since NMES effectiveness for neuromuscular rehabilitation is proportional to the torque evoked during training^2^, our results advocate against the use of TENS during or before a session of NMES as NMES training-induced increases in torque may be blunted. The possibility to amplify WPHF NMES-evoked extra torque by implementing stimulation protocols acting on the same pathways than high-frequency TENS, but in an opposite direction, remains to be investigated.
